# Chronic Cerebrospinal Venous Insufficiency Is Not Associated with Multiple Sclerosis and Its Severity: A Blind-Verified Study

**DOI:** 10.1371/journal.pone.0056031

**Published:** 2013-02-13

**Authors:** Maurizio A. Leone, Olga Raymkulova, Paola Naldi, Piergiorgio Lochner, Laura Bolamperti, Lorenzo Coppo, Alessandro Stecco, William Liboni

**Affiliations:** 1 MS Centre, Head and Neck Department, AOU Maggiore della Carità, Novara, Italy; 2 Interdisciplinary Research Center of Autoimmune Diseases, Novara, Italy; 3 Department of Neurology, Hospital Tappeiner, Merano, Italy; 4 SCDU Neurologia, Head and Neck Department, AOU Maggiore della Carità, Novara, Italy; 5 Istituto di Radiologia Diagnostica e Interventistica, AOU Maggiore della Carità, Novara, Italy; 6 SNC Neurology, Gradenigo Hospital, Torino, Italy; and Fondazione “Un passo insieme,” Valdellatorre, Italy; Research Inst. of Environmental Med., Nagoya Univ., Japan

## Abstract

**Background:**

Chronic Cerebrospinal Venous Insufficiency (CCSVI) has been associated with multiple sclerosis (MS) with a risk ranging from as high as two-hundred-fold to a protective effect. However, not all studies were blinded, and the efficacy of blinding was never assessed.

**Objective:**

To evaluate the association of CCSVI with MS in a cross-sectional blinded study and look for any association of CCSVI with the severity of MS.

**Methodology/Principal Findings:**

The Echo-color Doppler examination was carried out in accordance with Zamboni’s five criteria in 68 consecutive MS patients and 68 healthy controls, matched by gender and age (±5 years). Four experienced neurosonologists, blinded to the status of cases and controls, performed the study and were then asked to guess the status (case or control) of each participant. The number of positive CCSVI criteria was similar in the two groups. CCSVI, defined as the presence of two or more criteria, was detected in 21 cases (30.9%) and 23 controls (33.8%), with an OR of 0.9 (95%CL = 0.4–1.8, p = 0.71). The prevalence of CCSVI was related to age in cases (OR increasing from 0.2 to 1.4), but not in controls. CCSVI positive (N = 21) and negative (N = 47) MS patients were similar in clinical type, age at disease onset, disability, and fatigue. Disease duration was longer (16.5±9.8 years) in CCSVI positive than negative patients (11.5±7.4; p = 0.04). The operators correctly guessed 34/68 cases (50%) and 45/68 controls (66%) (p = 0.06), indicating a different success of blinding.

**Conclusions/Significance:**

CCSVI was not associated with MS itself, nor its severity. We cannot rule out the possibility that CCSVI is a consequence of MS or of aging. Blinding of sonographers is a key point in studying CCSVI and its verification should be a requisite of future studies.

## Introduction

Association of Chronic Cerebrospinal Venous Insufficiency (CCSVI), a recently proposed vascular condition, with multiple sclerosis (MS) was postulated in a study using extracranial and transcranial color-Doppler sonographic examination [1], whereas subsequent studies have produced conflicting results, and even questioned the existence of CCSVI itself [2]. By contrast with the original report, in which CCSVI was present in all the cases and none of controls, a very uncommon finding for a diagnostic procedure, the results have been amazingly variable with a risk ranging from as high as two-hundred-fold to even a protective effect [3], [4], [5], [6], [7], [8], [9], [10]. This was presumably due to technical and physiological factors and difference in expertise [1], [11]; however, any factor should apply to both cases and controls, and therefore it is unlikely that this would affect the results of studies, provided that blinding is assured. Some of the studies performed so far were not blinded and blinding efficacy was never assessed. Furthermore, many studies used only one sonographer; if he/she was able to recognize a subject’s status as case or control, this may also have affected blinding.

Thus we performed a cross-sectional blinded study to evaluate the association of CCSVI with MS, controlling the achievement of blinding. We used four sonographers to perform the echo-color-Doppler examination (ECD). Our secondary aim was to look for any association of CCSVI with the severity of MS.

## Methods

### Ethics Statement

The study was approved by the Ethical Committee of the “AOU Maggiore della Carità”, Novara (# 28/11). Informed consent, including the commitment to refrain from speaking with the examiners, was signed by all subjects.

### Cases

We prospectively enrolled all consecutive patients (age >18) attending our MS Centre (a first referral Centre that regularly follows about 700 patients) from March to September 2011 (N = 243). They were asked to participate in the study irrespective of the severity, duration or treatment of their disease. Those who accepted (N = 185) were listed and subsequently summoned for the ECD. Exclusion criteria were acute or chronic disabling disorders, severe cardiopathies or pulmonary diseases, prior cerebral or extracerebral venous thromboembolism, transient global amnesia, neoplasia, thrombophilic diseases, diabetes, head and neck surgery, vasculitides, family history of MS, cerebral vascular malformations, and congenital vascular malformations. Thirty MS patients were examined during the run-in period, 9 had one of the exclusion criteria, 3 refused, and 6 had the examination already performed at another Centre. Sixty-eight were included in this study, the others 69 are currently recruited in other ongoing studies. Comparison of the 68 cases with the 175 non included did not disclose any statistically significant difference for age, gender, place of birth, type of MS, treatment, age at onset, and disease duration (data not shown). All patients were visited and diagnoses were confirmed according to revised McDonald criteria [12]. Disease duration was measured from onset, defined as the first episode of focal neurological dysfunction indicative of MS. Patients with a relapse or using steroids during the previous 30 days were excluded. Demographic and clinical information included age, gender, age and symptoms at onset, Expanded Disability Status Scale (EDSS) [13], Kurtzke Functional Systems (FS) at onset [13], clinical events during the first year of disease, disease duration, number of relapses in the preceding 2 and 5 years, clinical course [14], and treatment. EDSS was performed on the same day as the ECD study. The Multiple Sclerosis Severity Score (MSSS) [15] was calculated for all patients and the Bayesian Risk Estimate for MS (BREMS) score [16] was calculated for Relapsing-Remitting (RR) and Secondary Progressive (SP) patients.

### Controls

Healthy controls, matched to cases by gender and age (±5 years) were selected from students, University personnel, relatives of patients admitted to the hospital for diseases other than MS, and their friends. Controls were visited to exclude a diagnosis of MS or other neurological diseases; those with a relative affected by MS were excluded. The exclusion criteria for the cases were applied to the controls.

### ECD Study

The ECD study was performed with a GE Vivid7 scanner, with a 7.5–10 MHz high-resolution linear array transducer for extracranial measurements and a 2–3 MHz probe for transcranial evaluation of venous drainage (GE Healthcare, Milwaukee, Wisconsin, USA). ECD was conducted initially in supine and afterwards in upright position after a short rest. The head was kept in line with the neck and in slight hyperextension. ECD evaluators examined flow characteristics of both the internal jugular vein (IJV) and the vertebral vein (VV) on the right side before, and the direction of flow in the deep cerebral veins (DCVs). The system settings were adjusted for the analysis of low-velocity signals; and the pulse repetition frequency was thus reduced to facilitate venous vessel detection. The subject’s head was held straight with appropriate head and arm supports to avoid venous compression. A large amount of ultrasonic gel was used and special care was taken to avoid compressing the neck. The ECD investigation was carried out in accordance with the five criteria suggested by Zamboni et al: [1]:

1. **Reflux in the IJVs and/or VVs in the supine and sitting position. Reflux in any vein >0.88 sec.** was considered ‘pathological.’ Flow was assessed during a period of apnoea following normal exhalation and not during Valsalva manoeuvre. The probe was located in a longitudinal and axial plane between vertebrae C6 and C7, which was maintained when participants changed to the upright position.
2. **Reflux in the DCVs.** Flow characteristics in at least one DCV were measured; flow in a reverse direction >0.5 s was considered ‘pathological’.
3. **High-resolution B-mode evidence of IJV stenosis or malformations (septum, valve malformation, flap, membrane, annulus). Stenosis was defined as a cross-sectional area (CSA) <0.3 cm^2^, measured at the thyroid gland (J2).**
4. **Flow not Doppler-detectable in one or both the IJVs or VVs.** following deep inspiration in supine and upright positions.
5. **Absence of physiological diameter increase of the IJV when passing from sitting to supine position.**

A subject was considered CCSVI-positive if ≥2 criteria were met.

### Procedure of the Study

Four neurosonologists participated in the study, two from our hospital and two from other hospitals. None of them works in a MS Centre. All of them have a long experience in neurosonology (range 10–30 years; 150–300 examinations/year). One (L.C.) was trained at Zamboni’s laboratory at the University of Ferrara. Two (L.B., W.L.) had a short 2-days training at the same laboratory, and the third (P.L.) was trained at another laboratory that uses Zamboni’s technique. During their training before this study, they examined 20–100 patients. During the run-in period at our Centre, the three sonographers with less CCSVI experience were tested for agreement to the fourth (L.C.), using eight patients or controls for each comparison.

Cases and controls were asked to fill the Fatigue Severity Scale (FSS) [17]. Blood pressure and heart rate were measured at the onset and end of the ECD. Much effort was directed to ensure blinding. Our MS Centre is situated in a building apart from the ECD laboratory. ECD operators were blinded to the status of cases and controls. An “outsider” (O.R.) was charged with the whole procedure, including blood pressure and heart frequency, FSS and EDSS evaluation, before entering the ECD laboratory. She transferred subjects to the laboratory, comfortably positioning them on a tilt chair and covering them with a blanket to conceal any hints such as injection marks potentially allowing for group assignment, and moved any aids out of the room. She alone was allowed to speak with the participants. Only at this point was the ECD operator allowed to enter the laboratory room. Subjects were instructed not to talk to the operators, and operators were not allowed to talk to them. The duration of the exam was established from the first to the last contact of the probe with the subject. The operators filled the study forms immediately after each examination, first indicating their guess on the status of each participant (case or control) and later the ECD features.

### Statistical Analysis

The sample size was calculated considering a 20% prevalence of CCSVI among controls as per the Buffalo study [18], with a power of 0.90 and an alpha of <0.05. It was thus estimated that 7 cases and 7 controls were required to achieve an OR of 43 (as in Zamboni‘s study), 35 and 35 for an OR of 4.3 (as in Buffalo study), and 64 and 64 for a more realistic OR of 3.0. Agreement was evaluated through the percentage agreement and Kappa (K) statistics [19]. Comparisons between groups were assessed using parametric (chisquare and chisquare for trend tests, Student’ test) and non-parametric methods (Kruskal-Wallis and Mann-Whitney U tests) where appropriate (deviation from normal distribution according to Shapiro-Wilk test). Data were analyzed with SAS [20] and R [21]. Blindness was evaluated with the Bang Index [22].

## Results

We included 68 patients with MS and 68 age- and gender-matched control individuals (age, p = 0.23). Most patients had RR-MS (N = 48, 71%), 15 were diagnosed with SP-MS (22%), and only 5 with PP-MS (7%). Demographic and clinical characteristics of MS types and controls are shown in table 1. The kappa values of the 3 less experienced operators versus the fourth were 0.14 (95% CL = −0.5 to 0.7) for two of them, and 0.33 (−0.4 to 1.1) for the third. The examination time ranged from 40 to 80 minutes.

**Table 1:** Clinical and demographic data of multiple sclerosis (MS) patients and healthy control subjects.

Characteristic	Relapsing-remitting MS	Progressive MS (secondary+primary)	All MS	Controls
Subjects, N	48	20	68	68
Age, years, mean (SD)	39.7 (10.2)	49.9 (8.0)	42.7 (10.6)	40.3 (12.5)
Gender, women/men	32/16	12/8	44/24	44/24
Age at onset, years, mean (SD)	29.0 (10.1)	30.9 (9.0)	29.7 (9.7)	
EDSS, median (IQ)	2.0 (1–2.5)	6.5 (6–7)	2.0 (1.5–6)	/
MSSS, median (IQ)	1.9 (1.0–3.3)	7.1 (5.9–8.1)	3.2 (1.4–6.0)	/
FSS, median (IQ)	40 (26–49)	53 (47–60)	44 (34–55)	/
Disease duration, years, mean (SD)	10.5 (6.6)	19.0 (9.6)	13.1 (8.5)	/
Patients on DMD or ISA, N (%)	27 (56.2)	5 (25.0)	32 (47.1)	/

SD = Standard Deviation; IQ = Interquartile Range; EDSS = Expanded Disability Status Scale; MSSS = Multiple Sclerosis Severity Score; FSS = Fatigue Severity Scale; DMD = Disease Modifying Drugs; ISA = immunosuppressive agents.

The prevalence of each Zamboni criterion among cases and controls is summarized in table 2. The most common abnormalities were criteria III and I, with no difference between cases and controls, as for the other three criteria. Criterion II was found in only one patient. The mean CSA was always higher in cases than controls, but after Bonferroni’s correction for multiple testing (six comparisons, p = 0.008) it never reached significance. For the supine position, it was 0.72 (SD = 0.62) in cases vs. 0.51 (0.45) in controls on the right side (p = 0.03) and 0.56 (SD = 0.33) vs. 0.43 (0.36) on the left side (p = 0.03). CSA in upright position was 0.20 (SD = 0.17) vs. 0.16 (0.17) on the right side (p = 0.26) and 0.18 (SD = 0.16) vs. 0.17 (0.27) on the left side (p = 0.79).

**Table 2:** Prevalence of CCSVI criteria in cases and controls (N, %).

	Cases(N = 68)	Controls(N = 68)	p	OR	95% CL
**Criterion I**	21 (30.9%)	20 (29.4%)	0.85	1.1	0.5–2.2
**Criterion II**	1 (1.5%)	0 (–)	0.32	–	–
**Criterion III**	32 (47.1%)	43 (63.2%)	0.06	0.5	0.3–1.0
**Criterion IV**	8 (11.8%)	4 (5.9)	0.23	2.1	0.6–7.5
**Criterion V**	7 (10.3%)	10 (14.7%)	0.44	0.7	0.2–1.9

The number of positive CCSVI criteria was similar in cases and controls (table 3). CCSVI, defined as the presence of two or more criteria, was present in 21 cases (30.9%) and 23 controls (33.8%), with an OR of 0.9 (95%CL = 0.4–1.8, p = 0.71). Its prevalence was similar for women (13/44 cases; 14/44 controls) and men (8/24 cases; 9/24 controls); the ORs for CCSVI were 0.9 (0.4–2.2, p = 0.82) for women, and 0.8 (0.3–2.7, p = 0.76) for men. The prevalence of CCSVI was related to age in cases, with the OR increasing from 0.2 to 1.4, but not in controls (table 4).

**Table 3:** Number of CCSVI criteria in cases and controls.

	Cases (N = 68)	Controls (N = 68)
3 criteria	4 (5.9%)	4 (5.9%)
2 criteria	17 (25.0%)	19 (27.9%)
1 criterion	23 (33.8%)	25 (36.8%)
0 criteria	24 (35.3%)	20 (29.4%)

p = 0.57 (chi-square for trend test).

**Table 4:** Prevalence of CCSVI criteria by age-groups (N, %).

	Cases (N = 68)	Controls (N = 68)	p	OR	95% CL
	Positive/Tot. (% pos)	Positive/Tot. (% pos)			
**<30 years**	1/10 (10.0)	7/19 (36.8)	0.12	0.2	0.1–1.8
**30–39**	5/17 (29.4)	5/14 (35.7)	0.71	0.8	0.2–3.4
**40–49**	7/22 (31.8)	4/15 (26.7)	0.74	1.3	0.3–5.5
**≥50**	8/19 (57.9)	7/20 (35.0)	0.65	1.4	0.4–4.9

p = 0.09 for cases and 0.80 for controls (chi-square for trend test).

We performed further analyses to evaluate whether CCSVI was related to clinical characteristics or disability in our MS sample. The prevalence of CCSVI was similar in PP (positive = 1/5, 20%), SP (5/15, 33.3%) or RR patients (15/48, 22%) (p = 0.85). We separated MS patients into 2 groups, according to ECD evidence of CCSVI (Table 5). Only disease duration was longer in CCSVI+ than in CCSVI- patients. Age at examination and at disease onset, EDSS, MSSS, BREMS, FSS, number of relapses in the preceding 2 and 5 years, and use of disease modifying or immunosuppressive therapy were similar between CCSVI+ and CCSVI- patients. We also compared the extremes of the MSSS distribution: CCSVI+patients were 5/12 in the first decile and 5/11 in the last 3 deciles (p = 0.79). The median MSSS was 3.6 (IQ range 1.5–5.5) in patients with no CCSVI criteria, 3.5 (1.7–6.2) in those with one criterion, 3.8 (1.7–6.4) in two criteria, and 4.0 (1.2–6.8) in three criteria (p = 0.98).

**Table 5:** Clinical features of CCSVI positive and negative patients.

Characteristic	CCSVI positive	CCSVI negative	p
Subjects, N	21	47	
Age, years, mean (SD)	45.2 (9.3)	41.6 (11.0)	0.17
Age at onset, years, mean (SD)	28.7 (9.2)	29.9 (10.1)	0.65
EDSS, median (IQ)	3 (1.5–6)	2 (1–6)	0.26
MSSS, median (IQ)	3.4 (1.7–6.4)	2.9 (1.4–5.7)	0.66
FSS, median (IQ)	47 (36–61)	44 (26–55)	0.35
Number of relapses in the preceding two years, median (IQ)	1 (0–2)	1 (0–2)	0.91
Number of relapses in the preceding five years, median (IQ)	3 (1–5)	3 (1–5)	0.83
BREMS score, median (IQ)	0.85 (0.34–1.63)	0.07 (−0.55 to 0.59)	0.11
Disease duration, years, mean (SD)	16.5 (9.8)	11.5 (7.4)	0.04
Patients on DMD or ISA, N (%)	9 (42.9)	23 (48.9)	0.09

SD = Standard Deviation; IQ = Interquartile Range; EDSS = Expanded Disability Status Scale; MSSS = Multiple Sclerosis Severity Score; FSS = fatigue Severity Scale; BREMS = Bayesian Risk Estimate for MS; DMD = Disease Modifying Drugs; ISA = immunosuppressive agents.

Lastly, since agreement among the four operators was not satisfactory, we performed a sensitivity analysis in which the OR was calculated for each of the four operators: it ranged from 0.3 (0.1–3.6; N = 22), to 0.6 (0.2–2.8; N = 41), 0.9 (0.2–4.2; N = 32), and 1.6 (0.5–5.8; N = 41).

The efficacy of our blinding procedure was different between cases and controls. The operators correctly guessed 34/68 cases (50%) and 45/68 controls (66%) (p = 0.06). The Bang Index was 0 (−0.20 to 0.20) for cases, indicating a successful blinding, and 0.32 (0.14–0.51) for controls, a figure that indicates a lack of blinding.

## Discussion

We found no association of CCSVI with MS in this blinded study. Three CCSVI criteria were more frequent in cases and two in controls, although never reaching statistical significance. The number of positive criteria was similar between cases and controls. Case-control studies performed after the original one [1] yield ORs for CCSVI ranging from 0.3 to 243.7 [3], [4], [5], [6], [7], [8], [9], [10]. The reason for such different findings is not clear. Heterogeneity may depend on the technical aspects of ECD examination, such as pressure of the ultrasound probe applied during the examination, selection of reference points, definitions of abnormalities, pulse repetition frequency, wall filter, different ways to estimate cross-sectional area, measurement of reflux in axial versus longitudinal orientation, use of Valsalva manoeuvre, and haemodynamic versus anatomical measurement of stenosis [11], [23]. Other possible sources of variability include physiological factors (head position, hydration status, use of arm abduction to relax cervical musculature, respiration), type of instrument used, expertise of operators, and specific CCSVI training. Although some of the above factors might affect the prevalence of CCSVI, they all apply to both cases and controls, and therefore it is unlikely that they affect the measure of risk, provided blinding is assured.

By contrast, other important factors related to the design of the study must be considered as sources of variability: choice of control group, number of operators, and blinding of operators, patients, and evaluators. To better understand these issues, we categorized case-control studies comparing MS or CIS patients with healthy controls published after Zamboni’s first study in positive-statistically significant, positive-not statistically significant (probably underpowered), and negative (Table 6), considering the distribution of different variables. Studies that did not provide figures for CCSVI, but only for some single criteria are also included [30], [31].

**Table 6:** Characteristics of the case-control studies on CCSVI and MS.

Characteristic of the studies	Positive, statistically	Positive, not statistically	Negative (N = 6)
	significant (N = 8)	significant (N = 3)	[4], [5], [9], [28], [29], [30]
	[6], [7], [10], [18], [24], [25], [31], [32]	[8], [26], [27]	
Odds ratio for CCSVI, range	3.2–244.7	1.5–1.9	NE-0.3
Sample size of cases, mean; SD; range	98.1; 82.8; 16–289	36.3;41.4;10–84	31.2;14.8;18–56
Blinding of operators (N,%)	4 (67%), 2 unknown	2 (67%)	4 (67%)
Blinding of ECD lecturers (N,%)	1 (13%)	0/3 (–)	4 (67%)
Training at Zamboni laboratory (N, %)	2 (25%)	1 (33%)	0 (–)
Number of operators	1–2	1–3	1–2
Prevalence of SP+PP (%)	36% (7 studies)	25% (2 studies)	21%
Type of controls	1 gen pop, 7 HC	2 HC, 1 HC+OD	5 HC, 1 OD
Prevalence of CCSVI in cases, median, range;	42; 7–84 (7 studies)	20; 20–50	0;0–5 (4 studies)
Prevalence of CCSVI in controls, median; range.	8; 0–23 (7 studies)	25; 0–36	0; 0–5 (4 studies)

NE = not estimable; SD = Standard Deviation; HC = healthy controls; OD = other diseases;

Negative studies had a lower sample size, no specific training at Zamboni’s laboratory, and a lower prevalence of CCSVI in controls. This finding may reflect scanty experience of sonographers with this new entity, which has been questioned as the cause of negative findings [33], especially when sample size is low. In addition, authors declaring a training in Zamboni’s laboratory [10], [18], [26] were able to find CCSVI more frequently (6–36%) among controls than those not declaring such training [4], [5], [6], [7], [8], [9], [23], [24], [25], [26], [27], [28], [29], [32] (0–25%). Here too, however, scanty CCSVI experience would apply to both cases and controls and should not have affected the risk. Our negative study has a sample size higher than any previous negative study, all operators had a specific CCSVI training, and our prevalence of CCSVI in controls is intermediate between two other large studies [18], [25], thus making unlikely that our study is hampered by methodological problems leading to under-ascertainment of venous abnormalities.

The other key factor to be considered in a procedure such as venous ECD, itself subjected to a high degree of subjectivity and variation, is blinding. However, blinding may or not be successful, since many variables that could facilitate discernment of a participant’s status might not be known. To control for possible biases, we used several precautions. Firstly, all the procedure was conducted by an “outsider”, who was the only one allowed to converse with the participants. Secondly, we used four operators. While this could have increased the heterogeneity of the examinations, it may avoid the bias due to the skills of any single operator in recognizing a participants’ status. Thirdly, our sonographers were not in a position to identify patients and controls, since the MS Centre is far from the ultrasound laboratory, none of the operators works in a MS clinic, and two of them even came from other cities. Despite these precautions, they were able to identify controls, but not cases, in a percentage higher than by chance. The direction of this possible bias is not discernible, depending on their pre-conceived opinion in favor or against the association, which we do not know. Most previous studies declared blinding of the sonographers and described how it was sought, but none of them evaluated whether it was achieved. One way to overcome detection bias is to blind the reading of ECD. This was done in five studies [4], [5], [9], [10], [29], and it is interesting to note that all but one were negative. Thus the blinding issue in CCSVI studies needs to be better evaluated and blind reading of ECD should become the standard procedure.

We performed several analyses to seek a correlation of CCSVI with the clinical features of MS. Disease duration was longer in CCSVI+ than in CCSVI- patients, and age at ECD examination was related to the prevalence of CCSVI in MS patients, but not in controls. This may suggest that CCSVI is a consequence of the disease, as found by other studies [10], [18], though this would seem disproved by the lack of any correlation with its severity. Some case-controls studies [10], [18] found an association with progressive forms of MS and observational non-blinded studies claimed an association with MSSS [34], whereas others found findings similar to our’s [6], [25]. Thus this issue is still unclear and could depend on some unexplained factors related to disease duration and age. Alternatively a lower success of blinding in more severe patients could explain such difference; our sample was too small to perform such analysis. Fatigue was found associated with CCSVI in an open study [35], but we were not able to reply this finding in our sample.

Some limits of our study must be admitted: its sample was small and not designed to detect risks of marginal entity; agreement between the sonographers was poor, although the sensitivity analysis did not show any substantial difference in the ORs; and the blinding was unbalanced between cases and controls. Any other limitations apply to both cases and controls, and therefore are unlikely to affect our results.

In conclusion, our results point against a role of CCSVI in causation of MS, and in its severity. We cannot exclude the possibility that it is a consequence of MS or of aging. Blinding of sonographers and possibly of ECD readings is a key point in studying CCSVI and its verification should be a requisite of future studies. Even though CCSVI may not be linked to MS, carefully blinded studies merit future planning to ascertain risk factors for CCSVI and its possible association with other diseases. About 70% of the total cerebral blood volume is located in the venous bed and its variations in size and the possible damage to the vein blood-brain barrier might be associated with other pathological conditions.

## Acknowledgments

We acknowledge our MS patients and controls for their collaboration.
